
JOURNAL INFORMATION
==============================
NLM Title Abbreviation: ScientificWorldJournal
Journal ID: ScientificWorldJournal
NLM Journal ID: 101131163
Journal ID: TSWJ

The Scientific World Journal

ISSN: 2356-6140
EISSN: 1537-744X
Publisher: Wiley

ARTICLE INFORMATION
==============================
PMCID: PMC5900853
PMID: 17982587
DOI: 10.1100/tsw.2007.260
Article ID: 570374
Article version: 1
Subjects: Editorial

Decoding Drug Abuse in Noncoding RNA?

Wu Da-Yu *
Saterlee John S.
Genetics and Molecular Neurobiology Branch, Division of Basic Neuroscience and Behavioral Research, National Institute on Drug Abuse, National Institutes of Health, U.S. Department of Health and Human Services, USA
*Da-Yu Wu: wudy@nida.nih.govAcademic Editors: D. Shurtleff and S. Ferre

Publication date: 2007
Electronic publication date: 2007 Nov 2
Volume: 7
First page: 142
Last page: 145
Received 2007 Sep 7; Accepted 2007 Sep 7
Copyright: Copyright © 2007 Da-Yu Wu and John S. Saterlee.
Copyright year: 2007
License: This is an open access article distributed under the Creative Commons Attribution License, which permits unrestricted use, distribution, and reproduction in any medium, provided the original work is properly cited.
License URL: https://creativecommons.org/licenses/by/3.0/

==============================
Editorial
NIDA Special Issue on Frontiers in Addiction Research
*TheScientificWorldJOURNAL* (2007) 7(S2), 142–145
ISSN 1537-744X; DOI 10.1100/tsw.2007.260

TheScientificWorldJOURNAL
www.thescientificworld.com

### Decoding Drug Abuse in Noncoding RNA?

Da-Yu Wu* and John S. Satterlee

*Genetics and Molecular Neurobiology Branch, Division of Basic Neuroscience and Behavioral Research, National Institute on Drug Abuse, National Institutes of Health, U.S. Department of Health and Human Services*

**E-mail:** wudy@nida.nih.gov, satterleej@nida.nih.gov

*Received September 7, 2007; Accepted September 7, 2007; Published November 2, 2007*

---

### NONCODING RNAS AND THEIR FUNCTION

The human genome comprises approximately 3 billion nucleotide (nt) bases and only 2% of them code genes that are translated directly into proteins. With the advancement of microarray technology, the completion of human genome sequencing, as well as the near completion of sequencing of several other vertebrate genomes, it is clear that a far greater number of genes transcribed into ribonucleic acids (RNAs) are not translated into proteins[1,2,3,4]. These so-called noncoding RNAs (ncRNAs) include ribosomal RNAs (rRNAs), transfer RNAs (tRNAs), and small nuclear RNAs (snRNAs). Several newly discovered families of ncRNAs include the 21-nt microRNAs (miRNAs), small nucleolar RNAs (snoRNAs), small interfering RNAs (siRNAs), repeat-associated small interfering RNAs (rasiRNAs), and others[1,3,5].

Many ncRNAs are integrated into large complexes with proteins and likely other RNAs that lead to diverse biological reactions. With the exponential expansion of the list of newly discovered ncRNAs and ncRNA types, we are only at the beginning of understanding the function of ncRNA. However, emerging evidence suggests that at least one essential function of ncRNA is gene expression regulation, through, for example, RNA interference, gene silencing, DNA demethylation, chromatin remodeling, and gene activation[2,6,7,8,9]. X inactive specific transcript RNA (XIST RNA), for example, spreads in cis along the X chromosome and likely recruits gene silencing factors in the X chromosome[10,11]. In chromatin remodeling, transcription of HOTAIR, a 2.2-kb ncRNA, is found in HOXC locus, and represses HOXD locus in trans[9]. Perhaps the best-understood mechanism of ncRNA's function in gene silencing is the role of miRNA. miRNAs are single-stranded, 18–25 nt, small ncRNAs. The primary transcript of miRNA is first excised by a double-stranded, RNA-specific endonuclease called Drosha into pre-miRNA. Pre-miRNA is then processed by another double-stranded RNA, Dicer. miRNA negatively regulate mRNA translation by forming a ribonucleoprotein complex, RNA-induced silencing complex (RISC), and by base-pairing, binds with the 3'-untranslated region (3'UTR) of target mRNAs. It is suggested that, depending on the miRNA identity, RISC can either suppress the mRNA translation or promote mRNA degradation on binding to the target mRNA[2]. Remarkably, due to the small size of miRNA and partial complementary binding for targeting requirements, one miRNA can regulate hundreds of mRNAs, and more than one-third of human genes may be regulated by miRNAs[12,13].

### ncRNAS IN THE NERVOUS SYSTEM AND IN NEUROLOGICAL DISORDERS

There are abundant ncRNAs in the nervous system, with many specifically localized only in the nervous system[14,15]. These ncRNAs form complex networks that direct nervous system differentiation and

*Corresponding author.
©2007 with author.
Published by TheScientificWorld; www.thescientificworld.com development[15,16]. Some representative evidence on the roles of ncRNA has come from animal studies. For example, in *C. elegans*, miR-273 and lsy-6, two miRNAs, form a gene regulatory network that controls the cell fate of two taste neurons[17]. In *Drosophila*, some large ncRNAs display preferential expression during embryogenesis and tissue specificity, with required roles in sensory organ development[18,19].

Data from vertebrates on miRNA function are intriguing as well. Mutant mice, which are defective in the pre-miRNA processing gene known as *dicer*, are embryonic lethal[20]. On the other hand, in zebrafish, the *dicer* zygotic mutant from the maternal mutant survives, but displays severe malformation in most brain regions[21]. At the cellular level, deletion of *dicer* in differentiating embryonic cultures led to complete loss of dopaminergic neurons, as well as reduction in GABAergic neurons, two types of neurons that populate the cortex and form reward and motivation neural circuits in the brain. In *in vivo* observations, localized conditional deletion of *dicer* in mice caused the loss of dopaminergic neurons in the midbrain and resulted in phenotypes similar to Parkinson's disease[22]. Furthermore, important evidence from this study indicates that the suppression of a single miRNA, miR-133b, may be responsible for the *dicer* deletion phenotype. Recently, the importance of ncRNA in brain development was further highlighted by the identification of HAR1, a 118-nt ncRNA region. HAR1 is part of the ncRNA gene HAR1F that is specifically expressed in the Cajal-Retzius neurons in the developing human cortex. The temporal and spatial pattern of the expression of this ncRNA suggests a role in cortical neuron migration and lamination, and human brain specification[23].

A growing body of evidence suggests that ncRNAs are also richly expressed and play important roles in adult brain function[14,24,25,26,27]. For example, a brain-specific miRNA, miR-134, is found to localize to the synaptodendritic compartment of rat hippocampal neurons where it negatively regulates the size of the dendritic spines through the inhibition of the translation of a synaptic protein Limk1[27]. Since it is known that changes in dendritic local protein synthesis are required for synaptic modulation implicated in learning and memory[28,29,30], miR-134 may play a critical role in brain function and plasticity.

The abundance and functional significance of ncRNA in the brain also implies that abnormal activity of ncRNA will likely lead to brain disorders. Indeed, many of the neurological disorders and diseases have been suggested to involve ncRNA deficiency: miR-189 variation has been linked to certain types of Tourette's syndrome[31]; deletion of DGCR8, which encodes a component of the complex that processes miRNA in the brain, results in DiGeorge-syndrome-related learning disabilities[32]; and ncRNA regulation of some "risk" genes may underlie the diverse findings of genetic linkage studies of schizophrenia[32].

### ncRNA IN DRUG ADDICTION

As a specialized brain disorder as well, it will be no surprise in the future to find out that ncRNAs are heavily involved in substance abuse and addiction. The neural circuits important for learning, memory, and reward are also involved in substance use disorder (SUD), and ncRNAs play key roles in neuronal differentiation, migration, and synaptic formation of neurons in these neural circuits[22,27,34,35]. In addition, many other brain mechanisms of addiction, such as synaptic modulation, dopamine transporter (DAT) regulation, and the signaling pathways in addiction and withdrawal, likely involve ncRNA function[22,26,27,33,34,36]. The abused substances themselves, such as alcohol and cocaine, have been known to interact closely with events and partners of ncRNA, such as DAT, GABRA2, and BDNF[27,34,35].

Although research on the role of ncRNAs in SUD is just beginning, it is likely that more and more evidence will emerge that ncRNAs play a critical regulatory role in SUD. We know, for example, that exposure to cocaine elevates the cAMP levels and triggers acetylation of histones at the loci of fosB promoter through the CREB-binding protein response[37]. Remodeling of chromatin sometimes requires ncRNA function[7,9]. With chronic cocaine use, deltaFosB is activated, which initiates coding and noncoding RNA transcription, and the resulting ncRNAs interact with the ncRNA for dopamine transporter (DAT) expression[35]. DAT plays key roles in the neuropharmacological and reinforcing effects of cocaine. It is also possible that drugs of abuse, such as cocaine, lead to synaptic plasticity through activation of miR-134 at the dendritic spines, up-regulate Limk1 protein translation, resulting in the growth and changes of dendritic spines, and, in turn, neurotransmission[27]. These ncRNA changes may, in part, lead to the drug-induced dendritic changes associated with SUD[37]. Alternative splicing of GABRA2 ncRNA can also bring about changes in GABA receptor gene expression, which is associated with addiction and withdrawal, and alcoholism[34,39].

Drugs of abuse can lead to cAMP activation, which stimulates the expression of MEG3 ncRNA, a maternal imprinting ncRNA highly expressed in the brain[36,40,41]. Affected ncRNA in imprinted embryos could affect cell proliferation, neuronal differentiation, and neural circuit formation, and increase the risk of tumor growth[41]. Although additional research is needed to uncover the precise and varied roles of ncRNA in SUD, these findings suggest that ncRNAs are involved in SUD, and will help to provide a better understanding of the genetic mechanisms underlying drug abuse and addiction.

### REFERENCES

1. Carninci, P., Kasukawa, T., Katayama, S., Gough, J., Frith, M.C., Maeda, N., Oyama, R., Ravasi, T., Lenhard, B., Wells, C., Kodzius, R., Shimokawa, K., Bajic, V.B., Brenner, S.E., Batalov, S., Forrest, A.R., Zavolan, M., Davis, M.J., Wilming, L.G., Aidinis, V., Allen, J.E., Ambesi-Impiombato, A., Apweiler, R., Aturaliya, R.N., Bailey, T.L., Bansal, M., Baxter, L., Beisel, K.W. et al. (2005) The transcriptional landscape of the mammalian genome. *Science* **309**, 1559-1563.
2. Cao, X., Yeo, G., Muotri, A.R., Kuwabara, T., and Gage, F.H. (2006) Noncoding RNA in the mammalian central nervous system. *Annu. Rev. Neurosci*. **29,** 77–103.
3. Kapranov, P., Cheng, J., Dike, S., Nix, D.A., Duttagupta, R., Willingham, A.T., Stadler, P.F., Hertel, J., Hackermuller, J., Hofacker, I.L., Bell, I., Cheung, E., Drenkow, J., Dumais, E., Patel, S., Helt, G., Ganesh, M., Ghosh, S., Piccolboni, A., Sementchenko, V., Tammana, H., and Gingeras, T.R (2007) RNA maps reveal new RNA classes and a possible function for pervasive transcription. *Science* **316**, 1484-1488.
4. Plomin, R. and Schalkwyk, L.C. (2007) Microarrays. *Dev. Sci*. **10,** 19–23.
5. Zamore, P.D. and Haley, B. (2005) Ribo-gnome: the big world of small RNAs. *Science* **309,** 1519–1524.
6. Costa, F.F. (2005) Non-coding RNAs: new players in eukaryotic biology. *Gene* **357,** 83–94.
7. Bernstein, E. and Allis, C.D. (2005) RNA meets chromatin. *Genes Dev* **19**, 1635-1655.
8. Goodrich, J.A. and Kugel, J.F. (2006) Non-coding-RNA regulators of RNA polymerase II transcription. *Nat. Rev. Mol. Cell Biol.* **7,** 612–616.
9. Rinn, J.L., Kertesz, M., Wang, J.K., Squazzo, S.L., Xu, X., Brugmann, S.A., Goodnough, L.H., Helms, J.A., Farnham, P.J., Segal, E., and Chang, H.Y. (2007) Functional demarcation of active and silent chromatin domains in human HOX loci by noncoding RNAs. *Cell* **129,** 1311–1323.
10. Penny, G.D., Kay, G.F., Sheardown, S.A., Rastan, S., and Brockdorff, N. (1996) Requirement for Xist in X chromosome inactivation. *Nature* **397,** 131–137.
11. Plath, K., Fang, J., Mlynarczyk-Evans, S.K., Cao, R., Worringer, K.A., Wang, H., de la Cruz, C.C., Otte, A.P., Panning, B., and Zhang, Y. (2003) Role of histone H3 lysine 27 methylation in X inactivation. *Science* **300,** 131–135.
12. Ambros, V. (2004) The function of animal microRNAs. *Nature* **431,** 350–355.
13. Lewis, B.P., Burge, C.B., and Bartel, D.P. (2005) Conserved seed pairing, often flanked by adenosines, indicates that thousands of human genes are microRNA targets. *Cell* **120,** 15–20.
14. Krichevsky, A.M., King, K.S., Donahue, C.P., Khrapko, K., and Kosik, K.S. (2003) A microRNA array reveals extensive regulation of microRNAs during brain development. *RNA* **9,** 1274–1281.
15. Mehler, M.F. and Mattick, J.S. (2007) Non-coding RNAs in the nervous system. *J. Physiol.* **575,** 333–341.
16. Klein, M.E., Imprey, S., and Goodman, R.H. (2005) Role reversal: the regulation of neuronal gene expression by microRNAs. *Curr. Opin. Neurobiol*. **15,** 507–513.
17. Johnston, R.J., Jr., Chang, S., Etchberger, J.F., Ortiz, C.O., and Hobert, O. (2005) From the cover: microRNA acting in a double-negative feedback loop to control a neuronal cell fate decision. *Proc. Natl. Acad. Sci. U. S. A.* **102,** 12449–12454.
18. Hardiman, K.E., Brewster, R., Khan, S.M., Deo, M., and Bodmer, R. (2002) The bereft gene, a potential target of the neural selector gene cut, contributes to bristle morphogenesis. *Genetics* **161,** 231–247.
19. Inagaki, S., Numata, K., Kondo, T., Tomita, M., Yasuda, K., Kanai, A., and Kageyama, Y. (2005) Identification and expression analysis of putative mRNA-like non-coding RNA in Drosophila. *Genes Cells* **10,** 1163–1173.
20. Bernstein, E., Kim, S.Y., Carmell, M.A., Murchison, E.P., Alcorn, H., Li, M.Z., Mills, A.A., Elledge, S.J., Anderson, K.V., and Hannon, G.J. (2003) Dicer is essential for mouse development. *Nat Genet* **35**, 215-217.

21. Giraldez, A.J., Cinalli, R.M., Glasner, M.E., Enright, A.J., Thomson, J.M., Baskerville, S., Hammond, S.M., Bartel, D.P., and Schier, A.F. (2005) MicroRNAs regulate brain morphogenesis in zebrafish. *Science* **308,** 833–838.
22. Kim, J., Inoue, K., Ishii, J., Vanti, W.B., Voronov, S.V., Murchison, E., Hannon, G., and Abeliovich, A. (2007) A microRNA feedback circuit in midbrain dopamine neurons. *Science* **317,** 1220–1224.
23. Pollard, K.S., Salama, S.R., Lambert, N., Lambot, M., Coppens, S., Pedersen, J.S., Katzman, S., King, S., King, B., Onodera, C., Siepel, A., Kern, A.D., Dehay, C., Igel, H., Ares, M., Jr., Vanderhaeghen, P., and Haussler, D. (2006) An RNA gene expressed during cortical development evolved rapidly in humans. *Nature* **443,** 167–172.
24. Sempere, L.F., Freemantle, S., Pitha-Rowe, I., Moss, E., Dmitrovsky, E., and Ambros, V. (2004) Expression profiling of mammalian microRNAs uncovers a subset of brain-expressed microRNAs with possible roles in murine and human neuronal differentiation. *Genome Biol*. **5,** R13.
25. Smirnova, L., Grafe, A., Seiler, A., Schumacher, S., Nitsch, R., and Wulczyn, F.G. (2005) Regulation of miRNA expression during neural cell specification. *Eur. J. Neursci*. **21,** 1469–1477.
26. Ashraf, S.I., McLoon, A.L., Sclarsic, S.M., and Kunes, S. (2006) Synaptic protein synthesis associated with memory is regulated by the RISC pathway in Drosophila. *Cell* **124,** 191–205.
27. Schratt, G.M., Tuebing, F., Nigh, E.A., Kane, C.G., Sabatini, M.E., Kiebler, M., and Greenberg, M.E. (2006) A brain-specific microRNA regulates dendritic spine development. *Nature* **439,** 283–289.
28. Ouyang, Y., Rosenstein, A., Kreiman, G., Schuman, E.M., and Kennedy, M.B. (1999) Tetanic stimulation leads to increased accumulation of Ca2/calmodulin-dependent protein kinase II via dendritic protein synthesis in hippocampal neurons. *J. Neurosci*. **19,** 7823–7833.
29. Schuman, E.M., Dynes, J.L., and Steward, O. (2006) Synaptic regulation of translation of dendritic mRNAs. *J. Neurosci*. **26,** 7143–7146.
30. Bramham, C.R. (2007) Control of synaptic consolidation in the dentate gyrus: mechanisms, functions, and therapeutic implications. *Prog. Brain Res*. **163,** 453–471.
31. Abelson, J.F., Kwan, K.Y., O'Roak, B.J., Stillman, A.A., Morgan, T.M., et al. (2005) Sequence variations in SLITRK1 are associated with Tourette's syndrome. *Science* **310,** 317–320.
32. Shiohama, A., Sasaki, T., Noda, S., Minoshima, S., and Shimizu, N. (2003) Molecular cloning and expression analysis of a novel gene DGCR8 located in the DiGeorge syndrome chromosomal region. *Biochem. Biophys. Res. Commun*. **304,** 184–190.
33. Perkins, D.O., Jeffries, C., and Sullivan, P. (2005) Expanding the 'central dogma': the regulatory role of nonprotein coding genes and implications for the genetic liability to schizophrenia. *Mol. Psychiatry* **10,** 69–78.
34. Tian, H., Chen, H.J., Cross, T.H., and Edenberg, H.J. (2005) Alternative splicing and promoter use in the human GABRA2 gene. *Mol. Brain Res*. **137,** 174–183.
35. Fuke, S., Sasagawa, N., and Ishiura, S. (2005) Identification and characterization of the Hesr1/Hey1 as a candidate trans-acting factor on gene expression through the 3' non-coding polymorphic region of the human dopamine transporter (DAT1) gene. *J. Biochem*. **137,** 205–216.
36. Zhao, J., Zhang, X., Zhou, Y., Ansell, P.J., and Klibanski, A. (2006) Cyclic AMP stimulates MEG3 gene expression in cells through a cAMP-response element (CRE) in the MEG3 proximal promoter region. *Int. J. Biochem. Cell Biol.* **38,** 1808–1820.
37. Levine, A.A., Guan, Z., Barco, A., Xu, S., Kandel, E.R., and Schwartz, J.H. (2005) CREB-binding protein controls response to cocaine by acetylating histones at the fosB promoter in the mouse striatum. *Proc. Natl. Acad. Sci. U. S. A.* **102,** 19186–19191.
38. Robinson, T.E. and Kolb, B. (2004) Structural plasticity associated with exposure to drugs of abuse. *Neuropharmacology* **47(Suppl 1)**, 33–46.
39. Liu, Z.H. and Ikemoto, S. (2007) The midbrain raphe nuclei mediate primary reinforcement via GABA(A) receptors. *Eur. J. Neurosci*. **25,** 735–743.
40. Zhang, X., Zhou, Y., Mehta, K.R., Danila, D.C., Scolavino, S., Johnson, S.R., and Klibanski, A. (2003) A pituitary-derived MEG3 isoform functions as a growth suppressor in tumor cells. *J. Clin. Endocrinol. Metab.* **88,** 5119–5126.
41. Zhou, Y., Zhong, Y., Wang, Y., Zhang, X., Batista, D.L., Gejman, R., Ansell, P.J., Zhao, J., Weng, C., and Klibanski, A. (2007) Activation of p53 by MEG3 non-coding RNA. *J. Biol. Chem.* **282,** 24731–24742.

**This article should be cited as follows:**

Wu, D.-Y. and Satterlee, J.S. (2007) Decoding drug abuse in noncoding RNA?. *TheScientificWorldJOURNAL* **7(S2)**, 142–145. DOI 10.1100/tsw.2007.260.
